# Relaxing retinectomy and autologous retinal transplant for complex retinal detachment with macular hole: a case series

**DOI:** 10.1186/s40942-026-00843-0

**Published:** 2026-03-31

**Authors:** Rami Madani, Armin Wolf, Jad Madani, Michael F. Ward, Matthew Cohen, Ferris Bayasi, Tarek Alasil

**Affiliations:** 1 Western Michigan University Homer Stryker MD School of Medicine, Kalamazoo, MI USA; 2 Department of Ophthalmology, University Hospital Ulm, Ulm, Germany; 3 Department of Ecology and Evolutionary Biology, University of California, Los Angeles, 612 Charles E. Young Drive South, Los Angeles, CA 90095 USA; 4 Vitreoretinal Surgery, Retina Institute of California, Pasadena, CA USA; 5 Kresge Eye Institute, Detroit Medical Center, Detroit, MI USA

**Keywords:** Retinal detachment, Rhegmatogenous retinal detachment, Macular hole, Proliferative vitreoretinopathy, Autologous retinal transplant, Relaxing retinectomy

## Abstract

**Background:**

To describe the surgical techniques for transplantation of an autologous retinal graft in cases of chronic retinal detachment with proliferative vitreoretinopathy and full-thickness macular hole.

**Methods:**

Descriptive case series of three consecutive patients (1 woman and 2 men; ages 60, 60, and 61) who underwent pars plana vitrectomy with peeling of proliferative vitreoretinopathy membranes, followed by relaxing retinectomy, where a notch was created to harvest a full-thickness neurosensory retinal graft. Perfluoro-n-octane was injected to flatten the retinectomy edges and stabilize the graft, which was then separated from the retinectomy edge and gently mobilized under Perfluoro-n-octane using a finesse loop to plug the macular hole. Endolaser was applied to the retinectomy edge under Perfluoro-n-octane, followed by a controlled Perfluoro-n-octane –air exchange and silicone oil (5000 cSt) infusion.

**Results:**

Primary outcomes were anatomical macular hole closure and retinal reattachment; visual outcomes were assessed by postoperative visual acuity, exam and imaging. Initial visual acuity ranged from light perception to hand motion. Postoperatively, visual acuity improved from light perception and hand motion to 20/400 and 20/200; the retina remained attached, and the macular hole was successfully closed, with the autologous retinal transplant integrating into the surrounding retinal tissue.

**Conclusions:**

Autologous retinal transplant, coupled with a relaxing retinectomy, may present a promising strategy for closing macular holes and improving visual acuity in cases of complex retinal detachment with proliferative vitreoretinopathy. We show that this procedure can be performed unimanually, potentially reducing technical demands in such cases.

**Supplementary Information:**

The online version contains supplementary material available at 10.1186/s40942-026-00843-0.

## Background

Macular holes (MH) occur secondary to rhegmatogenous retinal detachment (RRD) in approximately 1–3% of cases [1]. The incidence varies with the type of detachment, with proliferative vitreoretinopathy (PVR) recognized as a notable risk factor [2, 3] In such cases, MH development may result from foveal stretching due to tangential tractional forces [4]. Intraretinal PVR-related traction and contraction of epiretinal membranes can worsen retinal deformation, further predisposing to hole development.

Surgical management of MH in the setting of RRD generally involves pars plana vitrectomy (PPV) with internal limiting membrane (ILM) peeling or the inverted ILM flap technique, combined with either intraocular gas or silicone oil tamponade. While these approaches are effective for many uncomplicated cases with good closure rates, [5] closure rates are lower in large and recurrent MH, particularly those associated with high myopia or severe PVR [6]. In these cases, adjunctive procedures such as autologous retinal transplantation (ART) may be considered [7].

ART has emerged over the past decade as a promising option for large or refractory MH (> 400 μm), offering an overall closure rate of 94% compared with 59.6% for ILM peeling and 90.8% for the inverted ILM flap technique [8, 9]. The procedure entails harvesting a peripheral retinal graft and placing it over the MH, where it acts as a scaffold to promote Müller cell proliferation and facilitate anatomical closure [10]. Several landmark studies have demonstrated the viability of this approach. Grewal et al. reported successful closure and improved visual acuity in 87.8% of patients with chronic, large MH using ART [11]. Thomas AS and Mahmoud TH reported marked improvements in visual acuity following ART for retinal detachment (RD) associated with PVR, both in cases with and without MH [12]. Similarly, other studies have described favorable outcomes with minimal complications, further establishing ART as a valuable technique in challenging cases [13, 14].

In cases complicated by PVR, consequent retinal shortening often necessitates a relaxing retinectomy (RR) to relieve traction and improve re-attachment rates [15, 16]. However, detailed reports on the use of ART with RR remain limited [17]. This case series adds to existing literature on ART by highlighting its concurrent use with RR in patients with MH and RRD complicated by PVR. While previous descriptions of ART for similar cases have relied on bimanual tissue manipulation or chandelier endoillumination, [12] we demonstrate that favorable anatomical and functional outcomes can be achieved using a streamlined unimanual approach. By describing our refined surgical approach and outcomes, we aim to expand understanding of the role ART can play in managing this complex subset of patients.

## Methods

### Surgical technique

Three-port 23-gauge pars plana vitrectomy (Stellaris Elite [Bausch & Lomb, Inc., Rochester, NY]) was performed with retrobulbar anesthesia and monitored anesthesia care. All procedures were performed unimanually with either the vertical scissors or the vitrector in one hand and the light pipe in the other. Indocyanine green dye solution (0.06% concentration) was delivered to the retinal surface to help with the PVR peel, using the end-grasping forceps (Grieshaber 23-gauge ILM forceps by Alcon when indicated).

A localized or circumferential retinectomy, preceded by endodiathermy, was then created using the vitreous cutter, tailored to the extent of retinal contraction or pathology. Endodiathermy was delivered to cauterize blood vessels at the edges of the graft site. The neurosensory retinal graft itself was harvested by the surgeon (T.A.) from the detached retinal tissue at a preselected peripheral donor site at the retinectomy edge, typically in the nasal or inferior quadrant, avoiding large vessels. This was performed using vertical scissors (DORC), with Perfluoro-n-octane (PFO; Perfluoron by Alcon) instilled over the posterior pole and beyond the arcades to stabilize the macula and flatten the retinectomy edge, and to secure stability of the transplant during the final stages of harvesting the graft and during the transfer of the graft to its intended site, at the MH, using a finesse flex loop (Grieshaber 23-gauge Finesse Flex Loop DSP by Alcon). The edge of the graft was held, if required, using end-grasping forceps (Grieshaber 23-gauge ILM forceps by Alcon). The finesse flex loop was utilized to delicately smooth the graft edges under PFO liquid to ensure apposition without folding. Following completion of the retinectomy, endolaser photocoagulation was applied along the retinectomy edge, including the harvest site, to ensure secure adhesions. A careful PFO fluid-air exchange was then completed, followed by silicone oil tamponade to maintain graft and retinectomy stability during the postoperative healing period. The patients were positioned face down postoperatively for one week. The surgical technique is further illustrated in the video link.

## Results

The primary outcome evaluated was attachment of the retina (confirmed by ophthalmic exam and ultra-widefield fundus imaging) and anatomic MH closure after ART (confirmed by optical coherence tomography [OCT]). Secondary outcomes were visual acuity measurements at various time points, and integration of the graft with restoration of the outer retinal bands, external limiting membrane (ELM) and ellipsoid zone (EZ) measured using OCT. These were evaluated during all follow-up visits using the Spectralis OCT (Heidelberg Engineering, Heidelberg, Germany).

### Case 1

A 61-year-old man with a history of RRD secondary to a giant retinal tear in the left eye was initially treated with vitrectomy, endolaser, and silicone oil tamponade. Following silicone oil removal, an inferior star fold with PVR was observed, and sulfur hexafluoride (SF6) gas was used as a temporary tamponade.

The patient later presented to our service with recurrent inferior RD and a mature cataract in the left eye. Visual acuity was light perception, and the posterior segment was not visible. B-scan ultrasonography confirmed the diagnosis.

Phacoemulsification was performed to remove the mature cataract, followed by 23-gauge pars plana vitrectomy. Intraoperatively, a full-thickness MH (estimated to be between 1/2- and 2/3-disc diameter) and inferior PVR were noted. Despite extensive peeling of PVR membranes (using ILM forceps), the retina remained taut. Therefore, a 180-degree relaxing inferior retinectomy was pursued, where a notch was created inferiorly to harvest a full-thickness neurosensory retinal graft. PFO was injected to flatten the retinectomy edges and stabilize the graft, which was then separated from the retinectomy margin and gently mobilized under PFO with a finesse loop to cover the MH (Video 1). Endolaser was applied to the 180-degree retinectomy edge under PFO, followed by a controlled PFO–air exchange and silicone oil (5,000 cSt) infusion.

**Fig. 1:**
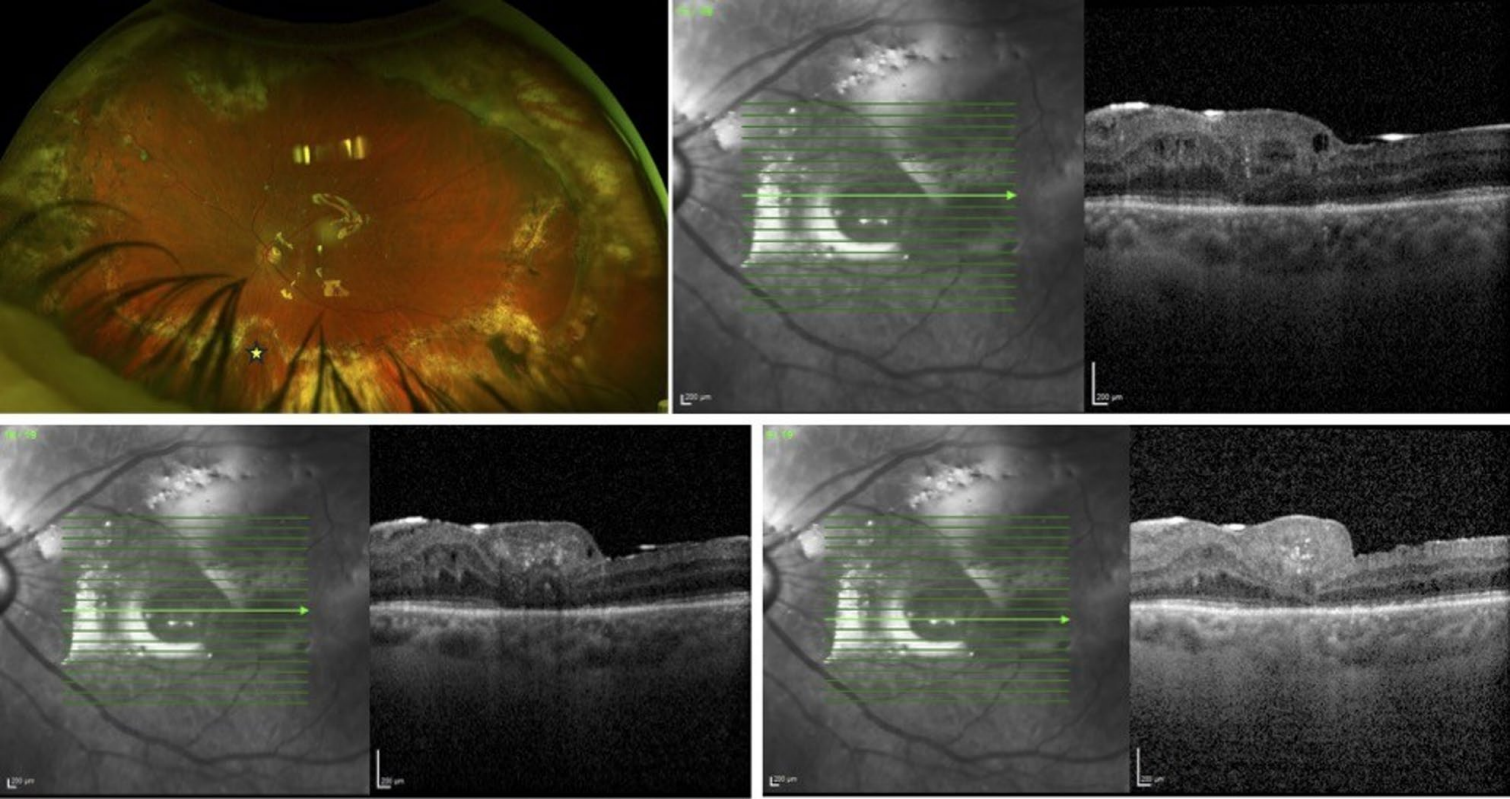
Postoperative ultra-widefield retinal imaging (Optos) and optical coherence tomography (Spectralis, Heidelberg Engineering) following repair of a recurrent inferior retinal detachment with proliferative vitreoretinopathy and macular hole (MH). Closure of MH was achieved using an autologous neurosensory retinal graft harvested from a notch (yellow star) within the inferior edge of a 180-degree relaxing inferior retinectomy. The retina is attached under silicone oil (5000 cSt) tamponade. Successful integration of the graft is visualized on OCT imaging. Visual acuity improved from light perception preoperatively to 20/400 postoperatively. Preoperative fundus photos and OCT images were not available due to advanced white cataract, and the diagnosis was confirmed using B-scan

Postoperatively, visual acuity in the left eye improved to 20/400+. The retina remained attached, and the MH was successfully closed with the autologous retinal transplant (ART), showing integration of the neurosensory graft into the surrounding retinal tissue (Fig. 1).

Five months postoperatively, the patient underwent silicone oil removal and placement of an anterior chamber intraocular lens (ACIOL). The retina subsequently redetached. Successful reattachment was achieved with fluid–air exchange, endolaser photocoagulation, and silicone oil (5000 cSt) infusion. Remarkably, the autologous retinal transplant remained viable following the redetachment and reattachment procedures.

### Case 2

A 60-year-old man with a history of RD repair and ACIOL implantation in the left eye presented with decreased vision (hand motion). Examination revealed a macula-off RD with a full-thickness MH (estimated to be between 1/2- and 2/3-disc diameter) and a retinal break along the superotemporal arcade.

A 23-gauge pars plana vitrectomy (PPV) was performed. PVR membranes were peeled from the detached nasal retina. During the creation of a nasal RR, a neurosensory retinal graft was harvested from the superonasal detached retina. PFO was injected to flatten the retinectomy edges and stabilize the graft, which was then separated from the donor site and gently mobilized under PFO using a finesse loop to cover the MH. Endolaser was applied at the retinectomy edge and around the retinal break at the superotemporal arcade. This was followed by a careful PFO–air exchange and silicone oil (5000 cSt) infusion.

**Fig. 2:**
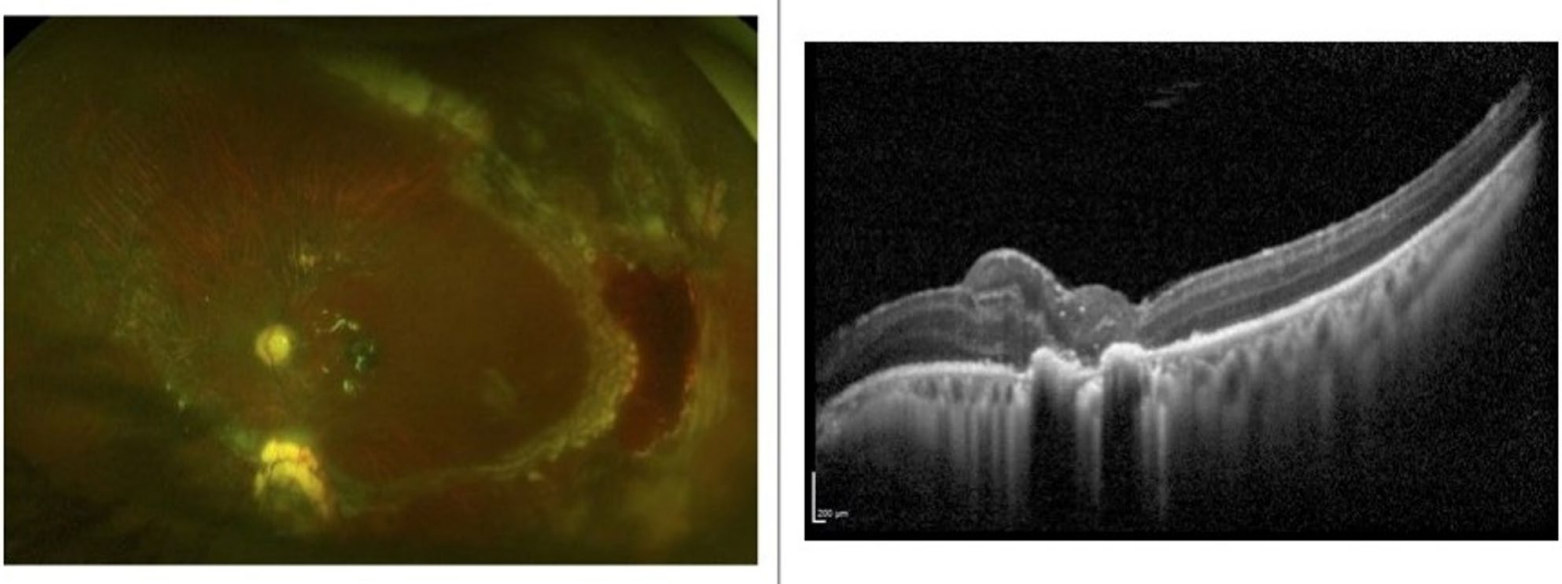
Postoperative ultra-widefield retinal imaging (Optos) and high-definition optical coherence tomography (Spectralis, Heidelberg Engineering) demonstrate the outcome following repair of a recurrent retinal detachment with proliferative vitreoretinopathy and a macular hole (MH). During creation of a nasal relaxing retinectomy, an autologous neurosensory retinal graft was harvested from the superonasal detached retina and maneuvered under perfluorocarbon liquid to plug the MH. A retinal break along the superotemporal arcade was identified intraoperatively and treated with endolaser photocoagulation. A 270-degree retinectomy was also completed and lasered. Mild hemorrhage noted at the temporal retinectomy edge resolved within one week postoperatively. The retina remains fully attached under silicone oil (5000 cSt) tamponade, and OCT imaging confirms successful integration of the graft

Postoperatively, visual acuity in the left eye improved to 20/200, the retina remained attached, and the MH was successfully closed with ART, which showed integration with the surrounding retinal tissue (Fig. 2).

Three months postoperatively, the patient underwent silicone oil removal. Four weeks later, the retina redetached but was successfully reattached using fluid–air exchange, endolaser photocoagulation, and silicone oil (5000 cSt) infusion. The autologous retinal transplant remained viable and structurally integrated following the redetachment and reattachment process.

### Case 3

A 60-year-old woman with a history of a refractory MH in the right eye, who had previously failed pars plana vitrectomy with ILM peel and gas tamponade as well as an attempt at ART, subsequently developed a RD with PVR. This was initially managed with scleral buckle, PVR membrane peeling, and silicone oil tamponade. Despite these interventions, the MH (intraoperatively estimated to be 1/2-disc diameter) remained open, and the nasal retina detached again with progressive PVR, and vision was counting fingers.

The patient underwent pars plana vitrectomy with additional PVR membrane peeling using ILM forceps, followed by a 120-degree relaxing nasal retinectomy. A notch was created nasally to harvest a full-thickness neurosensory retinal graft. PFO was used to flatten the retinectomy edges and stabilize the graft, which was then separated from the retinectomy margin and gently mobilized under PFO with a Finesse loop to cover the MH. Endolaser was applied to the 120-degree retinectomy edge under PFO, followed by a controlled PFO–air exchange and silicone oil (5000 cSt) infusion (Video 2). One year later, the RR was extended temporally and superotemporally.

**Fig. 3:**
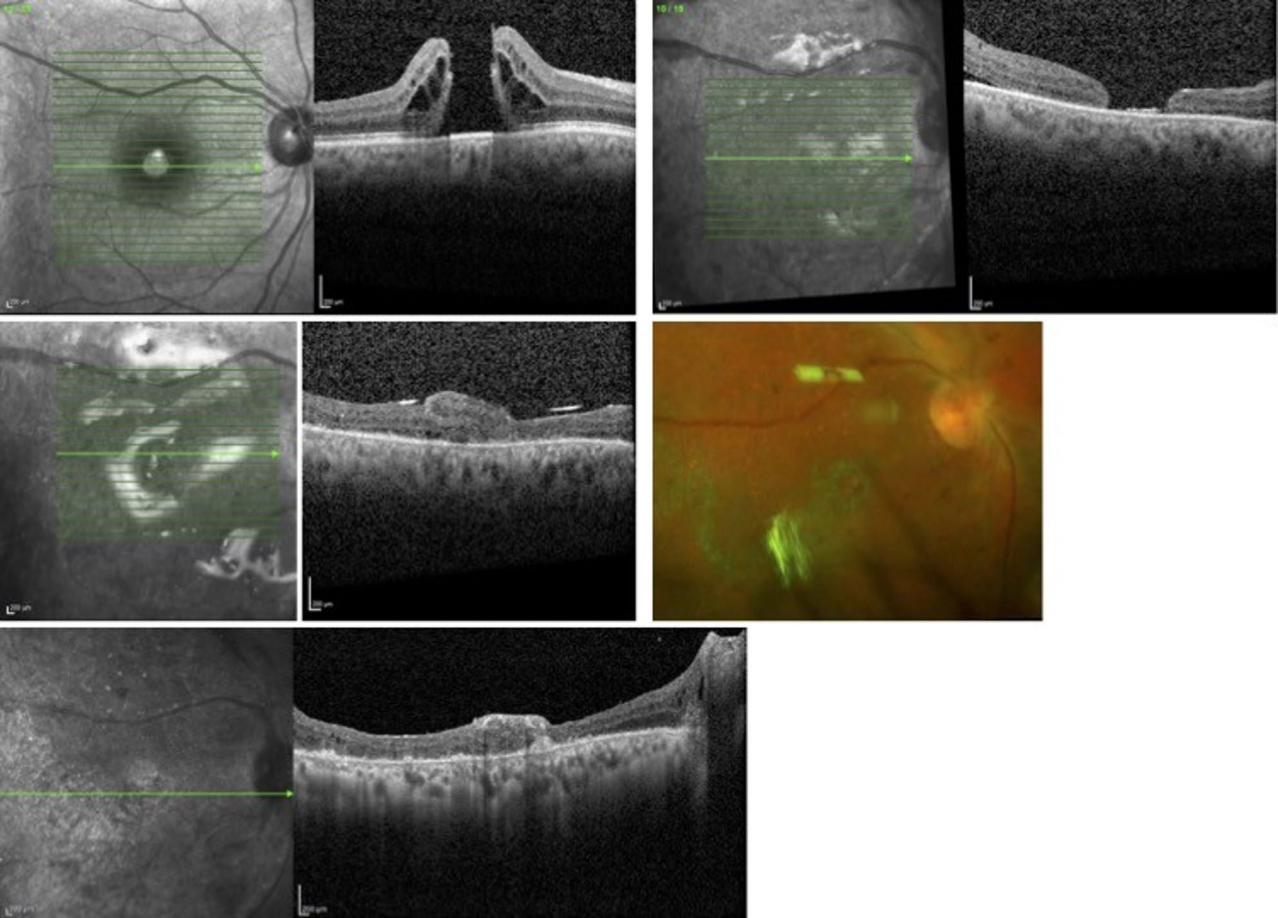
(Upper Row) Preoperative and postoperative optical coherence tomography (Spectralis, Heidelberg Engineering) scans illustrate a persistent macular hole (MH) following a failed autologous neurosensory retinal flap, complicated by retinal detachment and proliferative vitreoretinopathy (PVR). These were managed with scleral buckle placement, PVR membrane peeling, and silicone oil (5000 cSt) tamponade. (Middle Row) OCT macula and Optos images after additional PVR peeling and repeating autologous retinal transplantation (ART) demonstrate successful MH closure, with the graft well-positioned under silicone oil tamponade. (Lower Row) High-resolution OCT image after silicone oil removal shows ART in good position and integration with the surrounding retinal tissues

Postoperatively, visual acuity in the right eye improved to 20/400. The retina remained attached, and the MH was successfully closed with ART, and the neurosensory graft integrated into the surrounding retinal tissue (Fig. 3).

Twelve months postoperatively, the patient underwent removal of silicone oil. Intraoperatively, a localized nasal RD associated with a subretinal band was observed. The subretinal band was truncated and shortened, and the nasal retinectomy margins were refreshed, followed by fluid–air exchange, endolaser photocoagulation, and 18% SF₆ gas tamponade. The retina remained attached postoperatively, and the MH remained closed with ART. Subsequently, the patient’s visual acuity improved to 20/200.

## Discussion

In our case series, all three patients experienced postoperative improvement in visual acuity and sustained retinal reattachment. Specifically, visual acuity improved from light perception to 20/400+, from hand motion to 20/200, and from counting fingers to 20/200 across the three cases. Corresponding OCT scans demonstrated integration of the autologous retinal transplant with surrounding retinal tissue, supporting successful anatomical repair. While the degree of visual improvement varied across cases, all patients achieved measurable functional gain, suggesting the feasibility of combined ART and RR in the presence of concurrent PVR-associated RD and MH. In all three cases, the MH spanned the parafovea; therefore, hole closure and reattachment of the surrounding retina may help restore photoreceptor function in this region. Given the parafovea’s role in paracentral vision, this suggests that postoperative visual improvements could have largely been attributed to enhanced paracentral visual sensitivity [18]. In addition, patients reported reduced symptomatic burden after MH closure with ART, likely due to their relatively intrusive positive scotomas becoming negative [13]. While subjective descriptions were consistent, objective mapping of scotomas was precluded by the absence of microperimetry at our outpatient clinic. Nonetheless, proper graft placement was plausibly critical to the visual improvements described. Had the hole remained open, scotoma-related symptomatic burden likely would have persisted, and only marginal visual gain would have been expected, despite the successful management of PVR and retinal reattachment [19].

Most existing reports describe ART as a salvage option for large or refractory MHs, often in myopic or recurrent cases. Our series differs by pairing ART with RR, which was used to relieve PVR-related traction and allow retinal reattachment while harvesting donor tissue [15, 16]. This dual role may make RR a natural adjuvant to ART in these complex detachments, which is why we found it more logistically feasible than the transplantation of human amniotic membranes, a technique proposed by Rizzo et al. that can be used to plug large, refractory MH [20]. While prior work has demonstrated high rates of successful anatomical closure using this technique, the associated procurement costs and the technical challenges of inserting such membranes through small-gauge ports may limit its practical utility in complex cases [21]. Furthermore, while the amniotic membrane can serve as a scaffold to promote hole closure, functional outcomes may be theoretically constrained by its non-neurosensory nature [20].

## Anatomical factors affecting retinal graft integration and survival

The survival and structural integration of the autologous retinal graft depend on the integrity of the underlying retinal pigment epithelium (RPE) and the health of the surrounding retinal structures, including the nerve fiber layer. This presumably optimizes postoperative healing as the hole’s edges migrate centripetally to allow graft anastomosis with host retinal tissue [22]. Our series, as well as previous reports, suggests that slightly oversizing the graft relative to the MH may enhance visual outcomes [23, 24]. The integrity and thickness of the RPE–Bruch’s membrane complex can also have prognostic significance, further emphasizing the importance of RPE integrity in ART [25].

The ART graft layers harvested from the retinal periphery are comprised largely of rod cells and rod bipolar cells, in contrast with the cone-dense native macula. Nonetheless, following transplantation to the macula, bipolar and horizontal cell processes may extend their dendrites to form ectopic synapses with healthy photoreceptors outside the lesion (MH) [26]. These rod bipolar cell capabilities were observed by Beier C et al. in a rabbit model of retinal injury due to photocoagulation [27]. Analogous processes were histologically documented in night-blind (GNAT -/-) mice whose retinas were transplanted with rod precursors, where the regeneration of classic triad synaptic connections with bipolar and horizontal cells contributed to restored scotopic vision [28]. While the peripheral retinal graft inherently possesses lower cone density than the native macula, such neuronal plasticity may thus indicate the inner retina’s capacity to form new connections in humans, a process parallel to vascular responses. In particular, close approximation of the graft with the retinal defect can enhance angiogenic signaling, promoting vascular connections between host and transplanted retinal tissue. This vascular reperfusion is believed to support graft viability and facilitate the restoration of retinal tissue and function [29]. Furthermore, two of the three cases presented with detached retina after silicone oil removal, which could have resulted from insufficient retinal adhesion at the relaxing retinectomy margins [30]. Nonetheless, the ART grafts remained stable during secondary surgeries. This notion also seems to underline the integration of ART into the dehiscent retinal tissue at the transplantation site.

## Technique-related considerations of retinal graft placement and stabilization

There is a recognized surgical learning curve associated with harvesting and placing the neurosensory retinal transplant, particularly in eyes with detached retinas. Even though the neurosensory retina thins anteriorly at the equator, our study yielded positive outcomes irrespective of graft site. In cases 1 and 2, the graft was harvested from around the equator, while in case 3, it was obtained posterior to the equator. In the first two cases, the ILM was not peeled from the macula prior to graft placement, whereas in Case 3, the ILM had been peeled from the macula. Interestingly, graft thinning was not observed in Cases 1 and 2, suggesting that the remaining ILM around the MH may provide structural support to a thinner neurosensory transplant. However, the role of ILM status in graft survival and integration in the context of ART remains unclear, and further studies are needed to clarify its impact on postoperative outcomes in MH repair.

The method of graft stabilization was also critical to achieving successful postoperative outcomes in our series. Short-term tamponade with PFO proved valuable for stabilizing the graft and maintaining its orientation during transfer from the harvest site to the MH, and for improving oxygenation, particularly compared with silicone oil [31, 32]. To avoid subretinal entrapment, we directed the PFO jet away from the MH and donor site and ensured a careful PFO–air exchange prior to silicone oil infusion. These technical details minimized complications, and we observed no PFO entrapment in any of the patients.

Although the plane of the neurosensory retinal graft was more easily visualized due to the pre-existing RD, the final steps of harvesting the graft from around the superonasal equator of the left eye under PFO and mobilizing it toward the MH proved more technically demanding in Case 2. Graft transfer for similar PVR-complicated RD cases described by Thomas AS and Mahmoud TH, both with and without MH, additionally required chandelier endoillumination and bimanual tissue manipulation,[12] in contrast to our technique. The landmark study also used varying graft orientations, including a flipped placement for a PVR-complicated RD case without MH. We demonstrate that a standard upright orientation can be successfully integrated using a unimanual approach across all 3 cases. Therefore, this may indicate the potential reproducibility of our approach for patients with similarly complex presentations.

Lastly, in our cases of complex retinal detachment and ART in which long-term silicone oil tamponade was anticipated, we implanted a hydrophobic acrylic ACIOL (MTA3, Alcon, Fort Worth, TX, USA). This lens material is well tolerated in silicone-oil–filled eyes and helps prevent the opacification typically observed with hydrophilic IOLs [33].

### Limitations and future directions

Our study has several limitations due to its nature as a case series and exploration of an interesting surgical technique. With a small sample size, generalizability is limited. However, these were consecutive complex RDs with PVR and MHs that underwent combined RR with ART performed by a single surgeon. Larger-scale studies are needed to confirm the reproducibility of this approach in similar cases. Second, minimal linear and basal diameter measurements of the MH were missing due to the detached status of the retina. Third, microperimetry to provide fixation stability data in the 2- and 4-degree field was not available in our outpatient clinic to measure visual performance after ART and RD repair. Instead, we confirmed our results with visual acuity improvement, supported by OCT and ultra-widefield imaging.

Further investigation is needed to clarify the mechanisms of graft incorporation and patient-specific determinants of surgical success. It is important to elucidate long-term follow-up findings in these cases to improve surgical technique, notably through graft size optimization and minimal manipulation of the neurosensory retinal transplant tissue during transfer.

## Conclusions

Our experience suggests that autologous retinal transplant, coupled with relaxing retinectomy, may be a feasible strategy for closing macular holes in complex retinal detachment cases with PVR. We show that this technique can be performed without chandelier endoillumination or bimanual tissue manipulation.

## Supplementary Information

Below is the link to the electronic supplementary material.

Supplementary Material 1

Supplementary Material 2

## Data Availability

All data generated or analysed during this study are included in this published article and its supplementary information files.

## Abbreviations

MH: Macular holes
RRD: Rhegmatogenous retinal detachment
PVR: Proliferative vitreoretinopathy
PPV: Pars plana vitrectomy
ILM: Internal limiting membrane
EZ: Ellipsoid zone
ELM: External limiting membrane
ART: Autologous retinal transplant
RD: Retinal detachment
RR: Relaxing retinectomy
PFO: Perfluoro-n-octane
RPE: Retinal pigment epithelium
OCT: Optical coherence tomography
ACIOL: Anterior chamber intraocular lens
